# Ectopia cordis in an adult patient with COVID‐19: A case report and literature review

**DOI:** 10.1002/ccr3.5389

**Published:** 2022-02-06

**Authors:** Kamal M. Alshamiri, Abdulilah Z. Albriek, Tariq W. Farrag, Mostafa Q. Alshamiri

**Affiliations:** ^1^ 149994 Radiology Department King Saud Medical City Riyadh Saudi Arabia; ^2^ Cardiac Sciences Department College of Medicine King Saud University Riyadh Saudi Arabia

**Keywords:** adult ectopia cordis, congenital cardiac disease, COVID‐19 infection, critical cardiovascular disease

## Abstract

Ectopia cordis (EC) is a rare congenital condition characterized by a partial or complete defect of the anterior chest wall. It is associated with ventricular and atrial septal defects (ASD), Ebstein's anomaly, truncus arteriosus, transposition of the great vessels, tetralogy of Fallot, and hypoplastic left heart syndrome. This study aimed to explore the cardiac manifestations of EC complicated by coronavirus disease 2019 (COVID‐19). A 23‐year‐old male, born with EC, was admitted to the hospital for acute cough and fever. The patient was diagnosed with EC and ASD by computed tomography and COVID‐19 via a polymerase chain reaction swab test. Patients with ECs rarely survive till adulthood. However, due to the rarity of this syndrome, upon literature review, we did not find a case of EC with concurrent COVID‐19 infection. The patient underwent the required investigations and conventional treatment such as fluid resuscitation, antibiotics administration, and full code cardiopulmonary resuscitation. The interventions performed were unsuccessful, and the patient died. This case demonstrates a patient who lived with EC and its associated cardiac anomalies but died of COVID‐19 and its complications despite full resuscitation attempts. Our findings suggest that patients with EC may survive to adulthood if they have an incomplete EC, fewer intracardiac defects except for ASD, and an absence of an omphalocele.

## Graphical abstract

Ectopia cordis (EC) is a rare anomaly of unknown etiology rarely surviving to adulthood. We described EC who died of COVID‐19 and its complications despite full resuscitation. Patients with EC may survive to adulthood if they have an incomplete EC, fewer intracardiac, and an absence of an omphalocele.

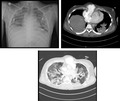

## 1 INTRODUCTION

During embryonic development, the cartilage bars of the sternum may fail to develop properly, leading to a sternal cleft. This results in a condition called ectopia cordis (EC), wherein the heart is outside the chest wall with either complete or partial pericardial coverage. It is extremely rare, and most patients do not survive till adulthood. Furthermore, an association of coronavirus disease 2019 (COVID‐19) infection with this condition has not been reported. However, a preexisting cardiovascular disease (CVD) or a viral complication on the heart has been known to increase the risk, mortality, and morbidity of COVID‐19.^1^, ^2^, ^3^, ^4^, ^5^, ^6^ This study aimed to present the clinical data of a patient with EC who survived till adulthood and got infected with COVID‐19. The study also reviewed relevant literature to explore the cardiac manifestations of EC complicated by COVID‐19.

## 2 METHODS

The review of literature was conducted utilizing databases from PubMed, ScienceDirect, and Google Scholar to ensure all relevant reports on EC have been included. Headings, subjects, and medical subject terms, as well as different combinations of basic search on EC, were used to search these databases regarding cardiac anomalies and COVID‐19.

Data were extracted if they met the inclusion criteria, which include the following:

1. Published scientific papers that focus on cases of EC
2. Systematic studies and medical case reports that are published in English
3. Associated cardiac anomaly and COVID‐19 infections.
4. Published data between 1925 and 2020.

All compatible data based on the inclusion criteria were reviewed and summarized by at least two independent reviewers.

## 3 CASE HISTORY, INVESTIGATIONS, TREATMENT, OUTCOME, AND FOLLOW‐UP

A 23‐year‐old male with EC presented to the emergency department complaining of shortness of breath, fever, and cough of 4‐day duration. He had a history of contact with a person, who was COVID‐19 positive.

**FIGURE 1:**
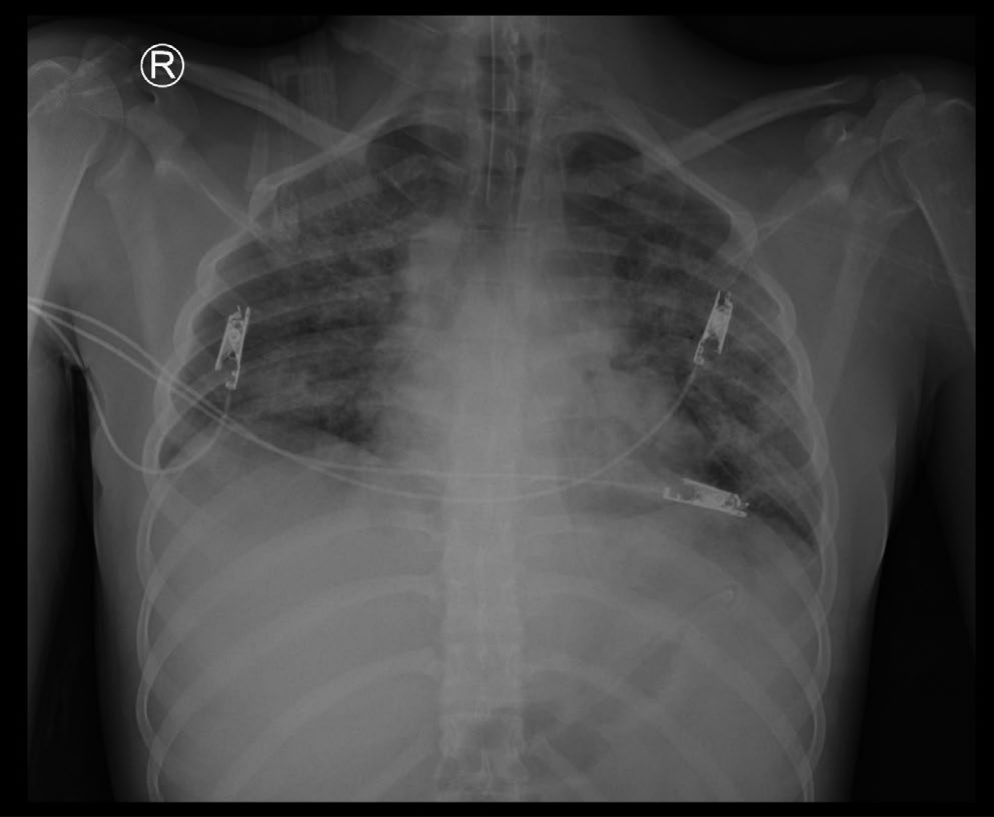
A frontal chest radiograph reveals bilateral patchy opacities throughout both lungs suggestive of ongoing infectious process

**FIGURE 2:**
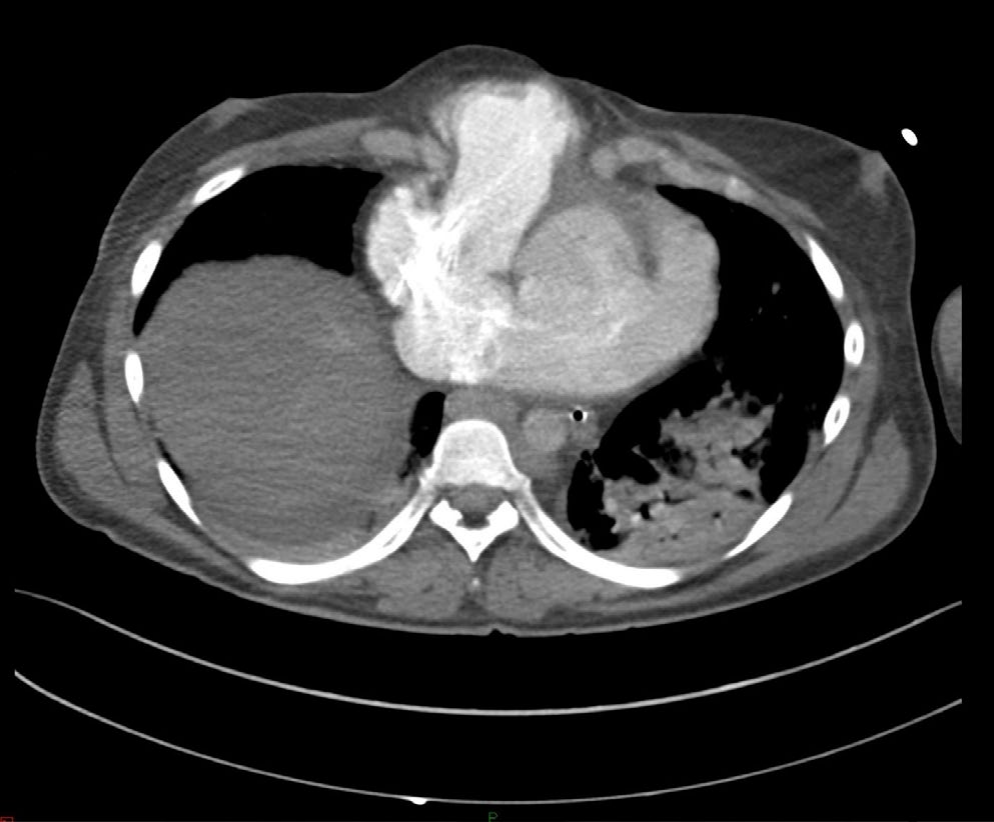
An enhanced computed tomography scan of the chest was performed to evaluate shortness of breath and chest pain. This shows partial ectopia cordis with right ventricular outflow tract herniation. The heart is seen extending through a midline defect in the sternum and lying outside the thorax, covered only by the skin. Atrial septal defect is also noted

**FIGURE 3:**
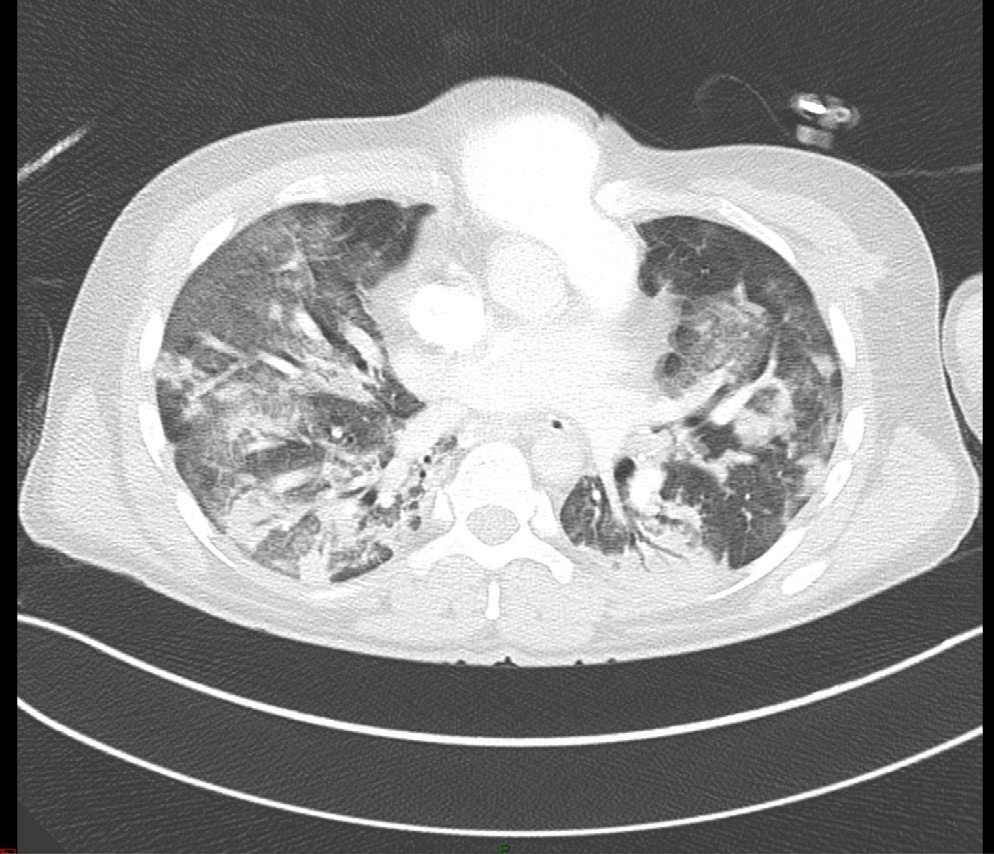
Lung window image of enhanced chest computed tomography scan reveals scattered ground‐glass opacities and patchy lung consolidation with the peripheral distribution. Viral infection was considered

Upon examination, the patient was febrile (38°C), generally ill with sinus tachycardia, central cyanosis, and oxygen desaturation on room air. Further examination revealed a bulged pulsatile mass covered with skin. Loud heart sounds and a systolic murmur were heard over the heart. An examination revealed coarse inspiratory crepitation all over the chest. The patient had a normal neurologic condition. The patient's condition deteriorated rapidly which required intubation and ventilation. Chest radiography revealed bilateral patchy opacities in both lungs in the background of questionable pulmonary edema (Figure 1). A bedside echocardiogram showed atrial septal defect (ASD), right ventricular dilatation, and normal left ventricular ejection fraction. A polymerase chain reaction (PCR) swab test was performed in addition to other laboratory workups. CT pulmonary angiogram was performed as pulmonary embolism was considered. It revealed no acute pulmonary embolism but found thoracic EC and secundum ASD (Figure 2). Ground‐glass opacities with peripheral distribution in computed tomography lung window image raise the possibility of underlying viral infection (Figure 3). The patient was admitted to the intensive care unit by the time the PCR swab tested positive. Troponin, creatinine phosphokinase, and renal profiles were increased. Despite full medical care with guidance from a local protocol for the care of patients with COVID‐19, the patient did not respond and continued to be hypotensive. He still had low oxygen saturation despite being on a mechanical ventilator. He remained febrile, with a temperature reaching 41°C and an increased renal function profile indicating multi‐organ involvement. He had a cardiac arrest requiring 10 min of cardiopulmonary resuscitation (CPR) until steady pulse was retained. He was administered high‐dose inotropic support, but 45 min later, he had another cardiac arrest requiring an hour of CPR. The patient was not able to recover and was declared dead.

The causes of death were postulated to be COVID‐19 infection associated with acute renal injury, acute respiratory distress syndrome, and septic shock.

## 4 DISCUSSION

Our case was a patient with a rare cardiac anomaly who acquired COVID‐19 from contact with a person who was confirmed to be COVID‐19 positive. He had contact with this person 10 days before presentation to the emergency department with symptoms compatible with COVID‐19. The patient succumbed within a few hours of the medical encounter with a cascade of oxygenation and renal function deterioration that ended in shock and cardiopulmonary arrest. Imaging excluded pulmonary thrombosis to explain the fulminant hospital course.

EC is a rare congenital defect with an estimated prevalence of 5.5–7.9 per million live births.^7^ The first report of EC was in 1706.^8^ The cause of EC is unknown, and most cases are sporadic and usually observed in newborns or children.^9^ Byron classified EC into four types: cervical, thoracic, thoracoabdominal, and abdominal.^10^ Thoracic and thoracoabdominal EC account for about 85% of the cases.^11^ Several congenital cardiac anomalies have been reported to be associated with EC in 82.2% of cases,^12^ including ventricular defect and ASD, Ebstein's anomaly, truncus arteriosus, transposition of the great vessels, tetralogy of Fallot, and hypoplastic left heart syndrome.^13^ ASD occurred in 53% of the cases.^14^ Our patient was documented to have ASD by CT angiography.

The heart can often be seen as a pulsating mass through the skin. In a partial EC, as seen in our patient, the heart is covered by the pericardium. Meanwhile, in a complete thoracic EC, the heart is displaced outside the thoracic cavity without a pericardial cover.^15^, ^16^

A few patients with pulmonary arterial hypertension and chronic thromboembolic pulmonary hypertension (CTEPH) suffer from COVID‐19. The case fatality rate of these patients was higher than that in the general population.^17^ However, in our case, there was no evidence of CTEPH on CT angiography.

The prognosis of thoracic EC is generally poor, and those who survive usually had fewer cardiac defects.^18^ The morbidity and mortality of EC are reduced when there is no concurrent omphalocele, such as in the case of our patient. An omphalocele is a sac containing organs, such as the bowel, liver, etc., that remains outside the abdominal cavity.^19^

There are no reports in the literature of adults with EC. Our patient may have survived until adulthood because his EC was only partial, had fewer intracardiac‐associated defects except for an ASD, and absence of an omphalocele.

Without a co‐existing COVID‐19 infection, the treatment for this condition would be surgical repair. The first repair for EC was attempted in 1925 by Cutler and Wilens.^20^ Koop (1975) reported the first successful repair of thoracic EC.^21^ In our case, our patient was in a severe condition due to the concurrent COVID‐19 infection. There was no time to manage the patient's cardiac anomaly.

In conclusion, EC and its associated anomalies are rare. Most patients do not survive to adulthood. Survival to adulthood is associated with fewer associated anomalies. However, a co‐existing COVID‐19 infection leads to a poor prognosis for any CVD and much more for complex cardiac anomalies such as EC. This report highlights the fact that EC may present with a clinical combination of spectral cardiac defects, and survival to adulthood may depend on the number of associated cardiac anomalies and having a partial rather than complete EC. The authors hope that this article sheds light on cases of patients with rare congenital cardiac anomalies who subsequently get infected with COVID‐19. This may help this special group of patients as we can ensure to provide them with early treatment and refer them to an advanced care facility to avoid death.

## CONFLICT OF INTEREST

The authors have no conflict of interest to disclosure.

## AUTHOR CONTRIBUTIONS

KMA: drafted the manuscript, collected the data, selected the figure legends, and revised manuscript. AZA: revised the images, confirmed the findings on the images, and ran the second review of the manuscript. TWF: collected the references, revised them, and indicated their correlation to the manuscript in addition to the manuscript revision. MQA: revised the manuscript, edited the manuscript for the journal requirement and language review, and confirmed the references related the manuscript.

## ETHICAL APPROVAL

The study was conducted at a tertiary care hospital in Saudi Arabia, with full authorization by the hospital. The patient's relative provided informed consent for the publication of this case. In the review of articles, the included studies were cleared with respect to the ethical handling of patients and related data.

## CONSENT

Written informed consent required for publication of this case was obtained.

## Data Availability

The data that support the findings of this study are available from the corresponding author upon reasonable request.
